# Chronotherapy and intervertebral disc degeneration: understanding the role of circadian rhythm in degenerative processes

**DOI:** 10.3389/fcell.2025.1586193

**Published:** 2025-06-03

**Authors:** Cong Zhang, Rende Zhang, Guanghai Zhao, Zuolong Wu, Wei Song, Rui Ran, Kaisheng Zhou, Haihong Zhang

**Affiliations:** ^1^ Department of Orthopedics, The Second Hospital and Clinical Medical School, Lanzhou University, Lanzhou, China; ^2^ Orthopaedics Key Laboratory of Gansu Province, The Second Hospital and Clinical Medical School, Lanzhou University, Lanzhou, China; ^3^ The Cuiying Biomedical Research Center, The Second Hospital and Clinical Medical School, Lanzhou University, Lanzhou, China; ^4^ Gansu Wuwei Hospital of Traditional Chinese Medicine, Wuwei, China

**Keywords:** intervertebral disc degeneration, circadian rhythm, chronotherapy, biological progress, BMAL-1

## Abstract

Intervertebral disc degeneration (IDD) and low back pain are prevalent issues globally, affecting a significant portion of the adult population. Recent research has highlighted the crucial role of circadian rhythms in the degenerative processes of intervertebral discs (IVDs). Circadian rhythms are regulated by a coordinated network of oscillators, consisting of a central clock system and various peripheral clock systems. These rhythms are influenced by environmental factors, particularly the light/dark cycle, and disruptions can lead to cumulative stress and imbalances within the body. The IVD tissue contains an autonomous oscillating peripheral clock, and evidence suggests that disruptions in these circadian rhythms can accelerate tissue aging and increase the risk of IDD. Studies have shown that reduced expression of clock genes, such as BMAL1, is an independent risk factor for IDD progression. Furthermore, circadian disruptions can imbalance anabolic and catabolic processes within IVDs, leading to tissue degeneration. Understanding the role of circadian rhythms in IDD may provide valuable insights into potential therapeutic strategies for preventing or mitigating disc degeneration. The review explores the entrainment of circadian rhythms with external physiological signals and their impact on disc physiology. Notably, disruptions in circadian rhythms have been linked to accelerated disc degeneration, with implications for tissue aging, metabolic imbalances, and inflammatory responses. Furthermore, the paper discusses potential therapeutic strategies, including chronotherapy, which aims to synchronize treatment interventions with circadian rhythms to optimize outcomes in IDD management. Understanding the intricate interplay between circadian rhythms and IDD could pave the way for innovative treatment approaches, ultimately improving patient care.

## 1 Introduction

Low back pain (LBP) and intervertebral disc degeneration (IDD) are widespread issues, affecting about two-thirds of adults and impacting approximately 568 million people globally (Clark and Horton, 2018; Cieza et al., 2021). Chronic low back pain is a significant cause of disability and mobility challenges, with its prevalence rising annually due to an aging population. This condition not only reduces work productivity but also contributes to substantial psychological stress and medical expenses, creating a considerable economic burden on society (Hurwitz et al., 2018). In the United States, the annual medical costs associated with chronic low back pain are estimated to be between $100 billion and $200 billion (Katz, 2006). Research indicates that up to 80% of individuals may experience chronic low back pain at some point in their lives, with prevalence rates ranging from 15% to 45% (Luoma et al., 2000). IDD is a key contributor to chronic low back pain, highlighting the urgent need for a deeper understanding and new treatment approaches (Vergroesen et al., 2015).

Circadian rhythms (CRs) disruption is influenced by multiple factors, primarily including environmental exposure, lifestyle habits, and genetic predisposition. Environmental disturbances are the leading cause—prolonged irregular light exposure, such as that caused by *social jetlag* and shift work, can directly impair the synchronization of the biological clock (Takahashi et al., 2018; Charlot et al., 2021). Lifestyle modifications are equally critical; in modern society, irregular sleep patterns, prolonged sitting, late-night eating, and skipping meals can disturb metabolic and endocrine functions, further exacerbating circadian misalignment (Kc et al., 2015). Notably, there exists a bidirectional relationship between rhythm disruption and mental health—individuals with depression and anxiety often exhibit circadian abnormalities, while circadian misalignment, in turn, aggravates psychiatric symptoms, creating a vicious cycle (Bromundt, 2014). In addition, genetic variation plays a crucial role in inter-individual differences; specific gene mutations may impair the core regulatory mechanisms of the circadian clock, resulting in abnormal sleep–wake cycles and heightened health risks (Jones et al., 2013).

CRs are intricately linked to the molecular mechanisms that govern various tissues, organs, and cells in the body (Fagiani et al., 2022). These biological clocks operate autonomously within the cells of different tissues, maintaining a dynamic balance that supports daily physiological functions (Fagiani et al., 2022). However, modern lifestyle factors and genetic mutations can disrupt these rhythms. Such disturbances in the circadian clock have been associated with a range of health issues, including cancer, cardiovascular diseases, neurodegenerative disorders, and dysfunctions of the liver, kidneys, and musculoskeletal system (Zhou et al., 2014; Wang et al., 2018; Amidi and Wu, 2022; Belloir et al., 2022). The role of inflammation as a key mediator linking circadian disruption to systemic diseases. One study has shown that systemic inflammation can disrupt CRs and alter neuroimmune dynamics. This research demonstrated that lipopolysaccharide (LPS)-induced systemic inflammation perturbed circadian gene oscillations in the hypothalamus, hippocampus, and liver, and triggered neuroinflammation, which could be linked to neurodegenerative diseases (Cheng et al., 2024). Furthermore, the interplay between CRs and inflammation is evident in cardiovascular diseases. Research has indicated that dysfunction of the circadian clock is implicated in the pathogenesis of cardiovascular disorders. For instance, in chronic kidney disease (CKD), altered circadian machinery in monocytes has been associated with cardiac inflammation and fibrosis. This is due to the altered expression of G protein-coupled receptor 68 (GPR68) in monocytes, which exacerbates inflammation and fibrosis in the heart under CKD conditions (Yoshida et al., 2021). The components of the musculoskeletal system, including bones, cartilage, and intervertebral discs (IVDs), also experience daily cycles of activity and rest, reflecting significant changes in CRs (Mei et al., 2024; Yu et al., 2022). For instance, mineral deposition in cranial bone tissue follows a rhythmic pattern with an average cycle of approximately 26.8 h (McElderry et al., 2013). Key elements involved in bone remodeling, such as calcium channels, disintegrins, metalloproteinases (MMPs), fibroblast growth factor (FGF), and Runt-related transcription factor (RUNX), display circadian patterns of gene expression (Kawai et al., 2014; Okada et al., 2020; Alford et al., 2015; Paulini et al., 2024; Tang et al., 2021; Kikyo, 2024). Additionally, the serum levels of hormones that regulate bone metabolism, including calcitonin, calcium, osteocalcin, parathyroid hormone C-terminal peptide, bone alkaline phosphatase, and tartrate-resistant acid phosphatase, also fluctuate throughout the day (Greenspan et al., 1997; Gundberg et al., 1985; Swanson et al., 2017). Research indicates that genetic models of circadian disruption are linked to a heightened risk of age-related musculoskeletal conditions, such as osteoporosis, osteoarthritis, tendinopathy, and IDD. Understanding these connections underscores the importance of maintaining CR integrity for musculoskeletal health (Gonçalves and Meng, 2019; Samsa et al., 2016). The role of inflammation as a key mediator linking circadian disruption to systemic diseases.

The role of CRs in IDD has garnered significant interest (Morris et al., 2021; Jiong Guo et al., 2022; Liu et al., 2022). The IVD is a highly rhythmic tissue that undergoes daily changes in loading patterns, influenced by the cycles of activity and rest. These fluctuations in loading lead to variations in osmotic pressure within the nucleus pulposus tissue (Malko et al., 1999). The resulting flow of interstitial fluid, driven by these osmotic changes, facilitates the exchange of nutrients and the removal of metabolic waste within the IVD (Zhang et al., 2020). Additionally, dynamic alterations in clock genes have been observed in human IVD cells, indicating an active relationship between CRs and disc health (Dudek et al., 2017). Notably, mice that lack the key clock protein BMAL1 exhibit early signs of degenerative changes in the IVD, highlighting the critical role of circadian mechanisms in maintaining the health and function of this vital tissue (Suyama et al., 2016).

While the exact molecular mechanisms by which CRs influence IDD remain unclear, growing evidence suggests that specific signals and cellular functions are integral to this regulation. Gaining a deeper understanding of these mechanisms is essential not only for uncovering the fundamental biological processes underlying IDD but also for developing innovative therapeutic strategies. These strategies could involve tailoring medication and optimizing surgical outcomes through adjustments to CRs. Moreover, since disruptions in CRs are linked to various factors that contribute to IDD, CRs may act as a comprehensive regulatory hub or core pathway. This could enable the control or delay of the degeneration process through multiple targets and pathways. In this review, we summarize recent findings related to CRs in both health and disease concerning the IVD. We emphasize the rhythmic functions of the IVD and explore how leveraging biological timing mechanisms can enhance tissue health and reduce degeneration. Our goal is to identify targeted chronotherapy strategies for IDD, which could offer new treatment ideas and approaches for spinal surgery.

## 2 Circadian rhythm and its regulatory mechanisms

### 2.1 Definition and characteristics of circadian rhythm

CRs are natural regulators found in nearly all forms of life, both eukaryotic and prokaryotic (Fagiani et al., 2022). They help synchronize physiological functions and behaviors in mammals, allowing them to adapt to environmental stressors and stimuli. These rhythms are aligned with the 24-h cycle of light and dark, as well as patterns of rest and activity and feeding and fasting. About 10% of human genes contain specific binding sites known as E-boxes, categorizing them as clock-controlled genes (CCGs) (Zhang et al., 2014). The fluctuations in these genes can influence the expression of up to 43% of related regulatory genes. Approximately 85% of human genes are clock controlled in at least one tissue type (van den Berg et al., 2017). Additionally, circadian clocks contribute to a 24-h rhythm in approximately 5%–20% of proteins in the body (Wang et al., 2017). It is important to note that the expression of these genes varies significantly across different tissues, highlighting the tissue-specific nature of these rhythms (Ko and Takahashi, 2006).

While the oscillation of mRNA transcription is a key output of the molecular clock, it is not the only one. The clock also regulates various cellular functions, including epigenetic changes (Liu XL. et al., 2024), the positioning of cellular components (Miyazaki et al., 2007), the rates of protein translation (Missra et al., 2015), and the management of redox processes and ion concentrations (Ko et al., 2009). Overall, circadian regulation is a complex system involving multiple molecular components that work together to ensure that an organism’s internal timing aligns with the external day-night cycle. This regulation is essential for maintaining homeostasis, coordinating bodily functions, and managing cellular activities at various levels (Cederroth et al., 2019).

### 2.2 Central and peripheral circadian clocks

The mammalian circadian clock functions as a coordinated network of oscillators, consisting mainly of a central clock system and various peripheral clock systems. The central clock is situated in the suprachiasmatic nucleus (SCN) of the hypothalamus. This nucleus plays a crucial role in receiving external light signals and transmitting synchronizing instructions throughout the body, ensuring that its rhythmic patterns align with the natural day-night cycle. As the primary pacemaker of CRs, the SCN relies heavily on light as its most potent synchronizer. When light enters the eyes, it is primarily detected by retinal ganglion cells, which transform light energy into electrical signals. These signals are then sent directly to the SCN and other specific regions of the brain. In response, the body generates various fluid, metabolic, and neural signals that act as messengers, helping to synchronize biological processes with the CRs (Chen et al., 2011).

Non-visual time cues, such as sleep-wake behaviors, preferences for sleep timing, and the stimulation of hormones like melatonin and serotonin, can reset the central clock located in the SCN (Astiz et al., 2019). This central clock system plays a vital role in regulating daily behaviors and physiological functions in mammals. It influences activity-rest cycles, sleep-wake patterns, daily fluctuations in blood pressure and body temperature, the secretion of melatonin from the pineal gland, and the release of hormones from the adrenal cortex (Moore and Klein, 1974; Moore and Eichler, 1976; Moore and Eichler, 1972). For instance, peak body temperature occurs in the early evening, which has been linked to increased carbohydrate utilization (Starkie et al., 1999). Hormone levels also fluctuate throughout the day, with testosterone, ghrelin, adiponectin, and cortisol. In contrast, hormones such as vasopressin, melatonin, leptin, thyroid-stimulating hormone, prolactin, growth hormone, and fibroblast growth factor are elevated at night (Gamble et al., 2014). These variations highlight the intricate relationship between the circadian clock and various physiological processes, ensuring that the body functions optimally according to the time of day.

The peripheral clock system is found in nearly all tissues and organs, including the heart, liver, skeletal muscle, tendons, joints, and IVDs (Costello et al., 2022). This system plays a crucial role in regulating metabolism, physiology, and behavior, with the expression patterns of clock genes exhibiting significant tissue specificity (Zhang et al., 2014). Research by Kenneth et al. has shown that in the musculoskeletal system, the skeletal muscle clock is responsible for regulating insulin sensitivity and metabolism, allowing muscles to adapt effectively to daily activity-rest cycles (Dyar et al., 2014). Additionally, the clock in tendons regulates protein secretion, facilitating the synthesis of collagen to repair fibers in high-load tissues (Chang et al., 2020). The peripheral clock integrates external time cues, contributing to the regulation of cellular functions. The rhythms of peripheral organs are synchronized by the central clock, which influences body temperature, hormonal rhythms, and the timing of behaviors such as food intake (Yoo et al., 2004; Guo et al., 2006). While the central clock does not directly control the oscillations of the peripheral clock, it does manage these peripheral oscillators through sympathetic and parasympathetic nervous regulation (Albrecht, 2012), endocrine signaling (Wu and Fu, 2017), and monitoring of eating and activity patterns (Schibler et al., 2003).

Circadian oscillations are present in cells throughout the central nervous system and most other body cells, with the peripheral clock being synchronized to the central clock located in the SCN. This synchronization drives the expression of core clock genes, leading to the production of CRs. It is currently understood that the SCN integrates temporal information, which is essential for synchronizing cellular biological rhythms within the same tissue and for maintaining specific time intervals between the biological rhythms of different tissues (Guo et al., 2006). However, the traditional linear model of “external environment - central clock - peripheral clock” is insufficient to explain the consistent oscillations of circadian clocks across mammals. This indicates the presence of a more sophisticated and complex “central-peripheral” interactive regulatory network (Kowalska and Brown, 2007; Husse et al., 2015). For example, research has shown that even after the specific knockout of 90% of BMAL1 in the forebrain/SCN of mice, the peripheral clock system continues to function, suggesting that peripheral clocks can operate independently to some extent (Izumo et al., 2014). The question of whether peripheral tissue clocks can feedback regulate the SCN and whether there is an interconnection between the rhythms of peripheral tissues remains an area of ongoing research.

### 2.3 Core molecular circadian clock

CRs persist even in the absence of environmental signals due to the body’s endogenous circadian oscillators, which drive daily rhythms at the molecular level (Patke et al., 2020). Peripheral tissues possess their own CRs capable of generating self-sustaining oscillations, leading to tissue-specific rhythms in gene transcription and translation. These rhythms are established through transcription-translation feedback loops (TTFLs). Cell-autonomous molecular circadian clocks are made up of intricate self-regulating TTFLs that control the rhythmic expression of downstream genes (CCGs) (Mohawk et al., 2012). CCGs play a crucial role in regulating the CRs of organisms and form complex regulatory networks through their interactions with other molecules. Despite the importance of CCGs, research on their specific mechanisms and how they regulate the physiological functions of various tissues and organs is still limited. Three distinct TTFLs have been identified that regulate the expression of CCGs, and these loops are essential for achieving the physiological functions of the circadian clock within specific organs or tissues. Further studies are necessary to elucidate the precise roles and regulatory mechanisms of each CCG, which will enhance our understanding of how CRs influence physiological processes across different biological systems, such as metabolic homeostasis, immune regulation, and neuroendocrine signaling.

#### 2.3.1 Core feedback loop

**FIGURE 1:**
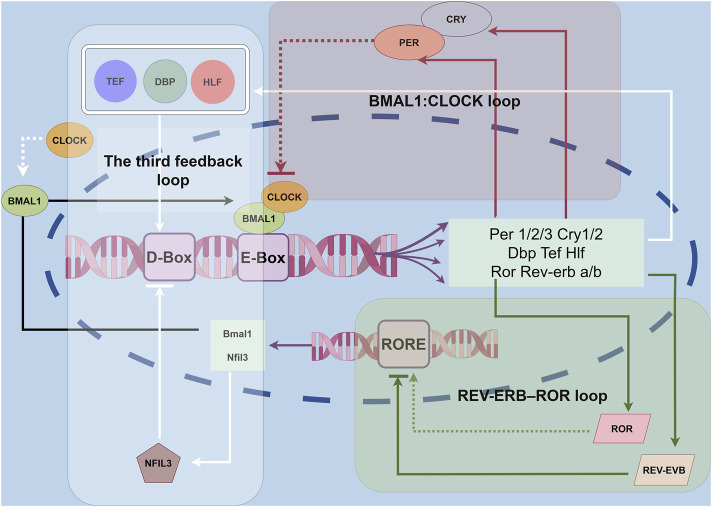
Molecular clocks in the IVD. The circadian clock is a network of transcriptional–translational feedback loops. The core feedback loop of the circadian clock begins when the BMAL1/CLOCK heterodimers bind to E-box elements in the promoter regions of target genes, including Per1/2 and Cry1/2. In the cytoplasm, PER1/2 and CRY1/2 form a multimeric complex, after which they translocate into the nucleus to inhibit the transcriptional activity of BMAL1/CLOCK. Additionally, nuclear hormone receptors such as REV-ERB and ROR form an auxiliary feedback loop that fine-tunes Bmal1 transcription. A third feedback loop involves the transcriptional activators DBP, TEF, and HLF, along with the repressor NFIL3, which rhythmically bind to D-box elements to modulate the expression of genes like Per1/2 and Cry1/2.

The BMAL1: CLOCK loop is a key component of the CR regulation. This loop features two essential proteins, brain and muscle Arnt-like protein-1 (BMAL1) and circadian locomotor output cycles kaput (CLOCK), which act as positive regulators (Lane et al., 2023). As the day begins, the genes for these proteins are activated, leading to the production of BMAL1 and CLOCK. These proteins then combine to form heterodimers that bind to specific regions in the DNA, promoting the expression of various target genes, particularly those involved in the negative regulation of the circadian cycle, such as the Period (Per1, Per2, and Per3) and Cryptochrome (Cry1 and Cry2) gene families (Morris et al., 2021). Throughout the day, the levels of the BMAL1/CLOCK heterodimer increase. Once the Period (PER) and Cryptochrome (CRY) proteins are produced in the cytoplasm, they can form complexes that return to the nucleus. In the nucleus, these complexes inhibit the activity of the CLOCK-BMAL1 dimer, thereby reducing the transcriptional activation of the PER and CRY (Lowrey and Takahashi, 2004). During the night, these negative regulatory proteins are partially degraded, allowing the circadian clock to reset and begin a new cycle. Additionally, the expression of clock-controlled genes follows a circadian pattern (Lech et al., 2016). However, the mechanisms that determine how different tissues achieve their specific CRs remain unclear and require further investigation (Figure 1).

#### 2.3.2 REV-ERB/ROR stabilizing loop

The CLOCK-BMAL1 complex plays a crucial role in regulating its own expression by inhibiting the BMAL1 gene. It does this by activating the transcription of the NR1D1 and NR1D2 genes, which produce the nuclear receptors REV-ERBα and REV-ERBβ (Preitner et al., 2002). The promoter region of the BMAL1 gene contains specific sequences known as Retinoic acid-related orphan receptor (RoR) response elements. These elements serve as binding sites for both REV-ERB and ROR proteins. RORα binds to these response elements to activate BMAL1 transcription. However, ROR proteins, including RORα and RORγ, compete with REV-ERB proteins for the same binding sites on the DNA. This competition inhibits the ability of REV-ERB proteins to regulate downstream genes, ultimately leading to an increase in BMAL1 expression (Zhang et al., 2015; Roenneberg and Merrow, 2016) (Figure 1).

#### 2.3.3 D-Box stabilizing loop

The transcriptional circuit regulated by the BMAL1-CLOCK complex features a unique structure known as a proline- and acidic amino acid-rich leucine zipper (PAR-bZIP). This structure primarily includes proteins such as D-box-binding protein (DBP), thyrotroph embryonic factor (TEF), and hepatic leukemia factor (HLF) (Mitsui et al., 2001). These proteins interact with specific DNA sequences called D-Box sites. D-Box is a DNA cis-acting element that can bind to these transcription factors, thereby affecting the transcriptional activity of CR genes. By analyzing the binding sites of DBP and E4BP4 in the mouse liver, it was found that the D-Box sequence plays a key role in the input and output of CRs. This regulatory mechanism not only affects the rhythmicity of gene expression but also responds to environmental stimuli, thereby resetting the circadian rhythm (Yoshitane et al., 2019). It remains unclear how DBP, TEF, and HLF feed back onto the core loop, although this may be mediated through interactions with the D-box element. Additionally, the circuit is influenced by the inhibitory nuclear factor interleukin-3 regulated (NFIL3). NFIL3 binds to the D-box sequence, exerting an inhibitory effect on the transcriptional activity of the circuit. Furthermore, NFIL3 itself is regulated by the REV-ERB/ROR feedback loop, highlighting the interconnected nature of these regulatory mechanisms within the CR system (Gachon et al., 2004) (Figure 1).

### 2.4 Circadian entrainment factors

Circadian entrainment is the process of aligning the body’s internal rhythms with external physiological signals, ensuring that the functions of specific tissues are synchronized with the environment and other bodily systems (Yu et al., 2022). This alignment is influenced by various external cues, including light and dark cycles, patterns of activity and rest, food-related behaviors, fluctuations in body temperature, social interactions, and noise levels (Morris et al., 2021).

Light is the most significant factor affecting CRs (Husse et al., 2015). Its timing, intensity, spectral composition, duration, and pattern can all influence the phase and amplitude of these rhythms (Bass and Takahashi, 2010). Individual responses to light can vary based on factors such as age, the type of CR, and genetic background (Perry et al., 2024). The SCN in the brain plays a crucial role in regulating melatonin (Mel) secretion in response to light exposure. Mel is a hormone that regulates sleep and wake cycles; morning light inhibits Mel production, promoting wakefulness, while light at night can suppress Mel, disrupting CRs and sleep patterns (van der Lely et al., 2015). Research has shown that exposure to bright light in the morning—between 2,000 and 10,000 lux for one to three hours—can significantly enhance sleep-wake cycles, improve mood, and boost cognitive function (Kanathur and Harrington, 2010).

In addition to light, non-light factors such as dietary habits (Gabloffsky et al., 2022) and physical activity (Vitale and Weydahl, 2017) also play a role in CRs. In animal studies, daily feeding has been identified as a CR regulated by a food-entrained oscillator (FEO), which imposes limits on how these rhythms can be adjusted (Luby et al., 2012). Irregular eating habits significantly impact circadian rhythms (Charlot et al., 2021). This is particularly true in anorexia, bulimia, and binge eating disorder, where circadian rhythm disruption is considered a significant pathological feature (Menculini et al., 2019). In addition, time-restricted feeding (TRE), as a strategy to limit the eating window, has been studied for the treatment of obesity. TRE, by aligning eating times with metabolic circadian rhythms, may improve metabolic health markers and could potentially serve as a therapeutic approach to combat metabolic diseases. Although the evidence remains inconsistent, restricting food intake to a fixed time window of 8–12 h each day can enhance insulin sensitivity, lower blood glucose, cholesterol, and triglyceride levels, and promote weight loss (Lages et al., 2024). Current evidence suggests that Time-Restricted Eating may help realign the biological clock and reduce the risk of metabolic diseases (Adafer et al., 2020). Furthermore, the composition of the diet—such as high-fat or ketogenic diets (Pickel and Sung, 2020)—and the type of CR (Takahashi et al., 2018) may also influence the expression of circadian clock genes.

## 3 The relationship between circadian rhythms and intervertebral disc physiology

### 3.1 The IVD is a tissue with diurnal rhythmic behavior

Diurnal rhythms refer to 24-h biological oscillations that are synchronized with external cues, such as light-dark cycles, while CRs denote endogenous oscillations that persist under constant environmental conditions. To avoid conceptual ambiguity, studies employing time-restricted feeding or light-entrained models are cited as evidence of diurnal regulation (Menculini et al., 2019), whereas experiments conducted under free-running conditions (e.g., constant darkness or light) or involving clock gene knockout models are explicitly classified as investigations of circadian mechanisms (Wang et al., 2022).

**FIGURE 2:**
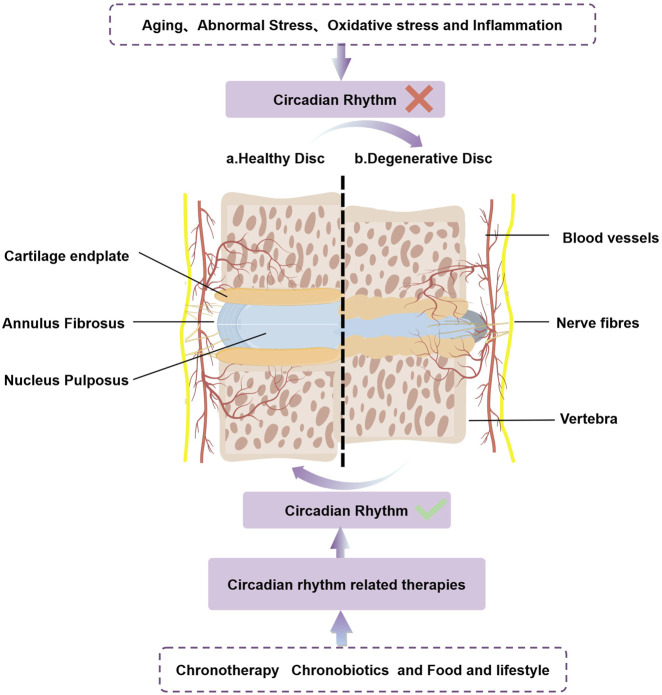
CRs play a crucial role in the normal/abnormal physiology of the intervertebral disc. Aging、abnormal stress、oxidative stress and inflammation accelerates IDD by disrupting intrinsic circadian rhythm. Circadian rhythm related therapies(chronotherapy、chronobiotics and food and lifestyle)can restore the damaged biological clock, slow the progression of IDD, and may even have the potential to cure IDD.

The IVD consists of three main components: the nucleus pulposus (NP), annulus fibrosus (AF), and cartilage endplate (CEP). The NP forms the core of the IVD and contains NP cells as well as essential extracellular matrix (ECM) components, including proteoglycan and collagen types I and II (Figure 2). Notably, the physiological functions of the IVD display diurnal characteristics, reflecting its adaptation to daily cycles. As one of the body’s primary load-bearing tissues, the IVD experiences significant compressive pressures, typically around 0.5 MPa while standing, and lower pressures during sleep (Massey et al., 2012). The loading patterns on the IVD change throughout the day, influenced by the rest and activity cycles (Ludescher et al., 2008). Research has shown that various properties of the IVD, such as height, water content, osmotic pressure, and mechanical characteristics, follow a CR (Reilly et al., 1984). During daytime activities, the spine endures increased pressure, causing interstitial fluid to flow through the AF and CEP towards areas of lower pressure. This results in a reduction of the intervertebral space, bulging of the annulus, and elevated osmotic pressure in the NP at the center of the disc. Conversely, at night, when the spine is relieved of pressure, the high osmotic pressure in the nucleus pulposus facilitates the return of interstitial fluid into the disc, restoring intervertebral height. This fluid movement is crucial for nutrient exchange and the removal of metabolic waste within the IVD, thereby maintaining the health and homeostasis of NPCs (Morris et al., 2021; Dudek et al., 2017; Haschtmann et al., 2006).

The metabolic processes within IVDs also exhibit a rhythmic pattern. Due to their aneural and avascular characteristics, IVDs are less responsive to traditional systemic time cues that are typically conveyed through nerves or hormones (Morris et al., 2021). Nutrients such as glucose and oxygen reach the IVD through two primary mechanisms: diffusion from the vertebral vascular supply, which ends at the CEP, and load-induced fluid convection that facilitates nutrient transport. Glucose, oxygen, and lactate are vital for the metabolism of IVD cells, and reduced transport of these nutrients is often linked to age-related disc degeneration. Furthermore, in cases of disc degeneration, such as NP herniation and the formation of fissures in the AF, the IVD’s response to daily mechanical load changes diminishes. This weakened responsiveness further decreases material circulation within the discs.

### 3.2 The IVD tissue contains an autonomously oscillating peripheral circadian clock

Recent research has increasingly highlighted the presence of a molecular clock within IVDs, with evidence suggesting that its disruption can accelerate tissue aging and increase the risk of IDD (Zhang et al., 2020). A study by Yasuaki et al. examined how passive smoking impacts the expression of clock genes in the IVD, revealing that core clock genes are actively expressed in these tissues. Their findings indicated a clear temporal sensitivity in the expression of factors regulated by the central clock across a 24-h cycle in the NP, AF and CEP of rats (Numaguchi et al., 2016). Further investigations by Suyama et al. identified the mRNA expression of BMAL1 and RORα, along with their protein products, in the NP of rats. HIF-1α, a crucial regulator of homeostasis in the IVD, can be activated by these clock-related factors, suggesting that they may influence the activity and stability of IVD cells during the process of IDD (Suyama et al., 2016). Genetic ablation of *Bmal1* in IVD cells abolished autonomous CRs, as evidenced by the complete loss of PER2Luc bioluminescence oscillations in IVD explants under free-running conditions and the absence of rhythmicity at single-cell resolution in both AF and NP regions. qRT-PCR analyses further revealed attenuated expression rhythms of core clock genes *Per2* and *Dbp*, underscoring BMAL1’s indispensable role in maintaining canonical clock function. Intriguingly, residual oscillations in *Timp4* were observed, likely mediated by non-canonical regulatory inputs such as mechanical loading or systemic signals; however, these exhibited erratic phase patterns distinct from BMAL1-dependent rhythms, highlighting the centrality of intact molecular clocks in sustaining robust circadian coordination (Dudek et al., 2017). The IVD clock is crucial for regulating various signaling pathways, including those involved in matrix homeostasis, repair, mitochondrial function, fatty acid metabolism, endoplasmic reticulum stress, and apoptosis (Dudek et al., 2017).

### 3.3 Indirect regulation of IVD clocks by the central circadian clock

Although IVDs lack direct neural or vascular connections to the central circadian pacemaker, diurnal hormonal signals and systemic circadian outputs—such as core body temperature rhythms and daily activity cycles (as detailed in Section 3.1)—may serve as endogenous time cues for peripheral clocks within IVD tissue. A key systemic cue is the robust diurnal fluctuation in circulating glucocorticoid levels, orchestrated by oscillators within the SCN, hypothalamus, and adrenal gland. Glucocorticoids act as potent zeitgebers for peripheral clocks by binding to glucocorticoid response elements (GREs) in the promoter regions of core clock genes, thereby modulating their transcriptional activity (Ota et al., 2020; Shimba and Ikuta, 2020). For instance, the core circadian gene *Per2* contains specific GRE binding sites (So et al., 2009), and circadian oscillations in IVDs have been shown to be responsive to the synthetic glucocorticoid dexamethasone in *ex vivo* culture models (Morris et al., 2021). In addition, parathyroid hormone (PTH), which itself displays circadian oscillation in serum levels, has been demonstrated to entrain cartilage clocks *in vivo* (Okubo et al., 2015). Age-related degenerative changes, such as sclerosis of the CEP, may impair nutrient and humoral factor diffusion into the IVD, potentially contributing to disrupted clock entrainment.

Interestingly, 12-h/12-h temperature cycles that mimic physiological daily fluctuations in core body temperature are sufficient to entrain circadian rhythms in multiple peripheral tissues in mice (Goh et al., 2016). Dudek et al. further demonstrated that temperature cycles—38.5°C for 12 h followed by 35.5°C for 12 hours—could induce antiphase oscillations (180° shift) between IVDs and articular cartilage, while also amplifying rhythm amplitude (Dudek et al., 2017; Dudek et al., 2016). Moreover, findings by Grace H. Goh et al. revealed that caloric restriction can modulate the phase of SCN clock gene expression by altering the body temperature rhythm (Goh et al., 2016).

### 3.4 The impact of CR disruption on IVD

Chronic disruption of internal CRs, along with daily metabolic needs, can lead to cumulative stress and imbalances within the body, potentially contributing to the early onset of age-related diseases (Neves et al., 2022). A prospective cohort study found that shift workers are at a higher risk of developing LBP over time (Christensen et al., 2021). Similarly, a cross-sectional study indicated that nurses working shifts report more musculoskeletal symptoms and an increased risk of LBP (June and Cho, 2011). Disruptions in CRs—caused by factors such as aging, shift work, and jet lag—are recognized as significant risk factors that can accelerate IDD and related conditions (Wang et al., 2022; Zhao et al., 2012). Further research analyzing 149 random clinical samples and eight paired samples from the same individuals identified reduced expression of the clock gene BMAL1 as an independent risk factor for the progression of IDD (Wang et al., 2022). Additionally, disruptions in circadian signals—such as fluid dynamics, abnormal mechanical loading, osmotic pressure, and temperature fluctuations—can interfere with the treatment processes of IVDs. This interference may create a vicious cycle that exacerbates tissue degeneration, highlighting the importance of maintaining CR integrity for overall spinal health (Morris et al., 2021; Zhang et al., 2020; Dudek et al., 2017; Ding et al., 2021).

The homeostasis of IVDs is closely tied to CRs (Gong and Zhang, 2021). Disruptions in circadian genes within the IVDs of mice, influenced by environmental factors and cytokines, can lead to an imbalance between anabolic and catabolic processes, accelerating tissue aging and degeneration (Dudek et al., 2017; Bekki et al., 2020). Chronic disruptions in these rhythms may interact with other risk factors, contributing to the development of IDD. In rats exposed to passive smoking, the expression patterns of genes such as *Per1, Per2, Cry1, Nr1d1*, and *Npas2* exhibited phase shifts and reduced amplitude. These changes are believed to result from shifts in the central circadian clock that regulate feeding and waking behaviors (Numaguchi et al., 2016). Additionally, in rats on a high-fat diet subjected to light/dark reversal protocols, disruptions in CRs were associated with a significant decrease in the content of glycosaminoglycans in the IVD matrix (Kc et al., 2015).

BMAL1 is crucial for maintaining the health of IVDs (Kondratov et al., 2006). When BMAL1 is disrupted or deleted, it results in a complete loss of CRs, making mice more vulnerable to IDD (Mei et al., 2022). Age-related IDD was also observed in conditional BMAL1 knockout mice (Dudek et al., 2017). At the same time, downregulation of Bmal1 results in reduced expression of downstream gene NRF2, which in turn increases inflammatory responses, oxidative stress, and cell apoptosis (Peng et al., 2022). However, the exact mechanisms by which BMAL1 influences IDD remain unclear, and it is still uncertain whether other factors regulate BMAL1 expression in NPCs. Recent proteomics and phosphoproteomics studies have identified that hsa-let-7f-1-3p, a microRNA cluster, directly targets the 3′untranslated region (UTR) of BMAL1. This targeting promotes apoptosis in NPCs, inhibits the synthesis of ECM, and increases ECM degradation, thereby accelerating the progression of IDD (Mei et al., 2022).

## 4 The role of circadian rhythms in intervertebral disc diseases

### 4.1 The role of circadian rhythm disruption in accelerating IDD

The molecular regulatory network underlying IDD remains incompletely understood. Several common triggers, including aging, oxidative stress, abnormal mechanical stress, and inflammation, can disrupt the biological clock of IVDs (Fagiani et al., 2022). Key components of this biological clock play a crucial role in regulating intracellular signaling pathways (Zhang et al., 2020). As a result, the disruption of the biological clock may serve as a significant mechanism through which external factors contribute to the development of IDD. Understanding this connection could provide valuable insights into potential therapeutic strategies for preventing or mitigating disc degeneration (Figure 2).

#### 4.1.1 CR and aging

Aging is currently recognized as the only definitive risk factor for the onset and progression of IDD (Teraguchi et al., 2017). As individuals age, the biological clock of the IVD becomes disrupted. Epidemiological studies indicate a positive correlation between age and the incidence of lumbar disc herniation (Kim et al., 2018). Moreover, disruptions in CRs can contribute to premature aging and the development of IDD. With aging, the circadian clock undergoes changes that result in a loss of precise temporal regulation (Bae et al., 2019). Both animal models and human studies show a decline in the robustness of CRs, characterized by reduced oscillatory amplitude and altered circadian phases (Bae et al., 2019). Research into IDD across different age groups reveals that disc cells in younger individuals experience oxidative stress during early stages of degeneration, while older individuals are more prone to endoplasmic reticulum stress. These stress responses are likely linked to changes in CRs, highlighting the complex interplay between aging, biological clocks, and disc health (Liu P. et al., 2024). Dysregulation of BMAL1 is a significant risk factor for age-related IDD. BMAL1 is crucial for the transcriptional control of the senescence program, particularly through its role in AP-1 dependent pathways (Jachim et al., 2023). It is associated with various senescence-related processes, including redox regulation, nutrient metabolism, and responses to genotoxic stress (Zhang et al., 2022). In fibroblasts derived from aged mice, the rhythmic expression of BMAL1 is altered, exhibiting prolonged cycles and delayed phases (Jachim et al., 2023). Furthermore, nutrient-sensing pathways that are influenced by aging demonstrate tissue-specific oscillations due to their direct interactions with core clock genes (Bass and Takahashi, 2010).

#### 4.1.2 CR and abnormal stress

The spine serves as the primary load-bearing structure of the human body, and abnormal mechanical stress is a prevalent external factor contributing to IDD (Molladavoodi et al., 2020). Mechanical signals are believed to play a role in regulating the homeostasis of IVDs by influencing their biological clock (Ding et al., 2021). While physiological levels of mechanical stress can promote anabolic metabolism, excessive or pathological mechanical stress can lead to apoptosis in both NP and AF cells, as well as catabolic processes in NPCs (Yurube et al., 2021). Excessive mechanical strain disrupts CRs *via* the RhoA/ROCK signaling pathway, which inhibits the expression of core clock proteins in NPCs, reduces ECM production, and diminishes cellular viability. This disruption increases the risk of IDD (Ding et al., 2021). Conversely, inhibiting the RhoA-ROCK pathway has been shown to effectively restore the damaged biological clock of the IVD, offering potential therapeutic benefits for disc degeneration (Wang et al., 2022). Moreover, mechanical loading exhibits its own biological rhythm within the IVD (Ding et al., 2021). Research by Dudek et al. demonstrated that daily mechanical loads within physiological ranges, along with corresponding changes in osmotic pressure, can reset the biological clock of the IVD. This highlights the importance of mechanical signals in maintaining the disc’s biological clock. The mechanoreceptor PLD2 located on the cell membrane is instrumental in linking mechanical stimuli to the mTORC2-AKT-GSK3β pathway, thereby mediating the regulation of the disc’s biological clock through mechanical signals (Dudek et al., 2023).

#### 4.1.3 CR and oxidative stress

The onset of IDD is closely linked to oxidative stress resulting from the accumulation of reactive oxygen species (ROS) (Chen et al., 2023; Ansari et al., 2020). This oxidative stress initiates a cascade of pathophysiological changes associated with IDD, including inflammatory responses, degradation of the ECM, cellular senescence, and apoptosis (Francisco et al., 2022; Chen et al., 2024). In degenerated IVD tissue, the formation of new blood vessels increases the supply of blood and nutrients to the disc, which can inadvertently enhance ROS production by nutrient-deficient disc cells, thereby triggering further oxidative stress responses (Francisco et al., 2022). There exists a bidirectional relationship between the cellular circadian clock and ROS. Disruption of CRs can negatively impact cellular redox metabolism, hinder ROS clearance, and result in abnormal ROS accumulation (Chen et al., 2023; Rabinovich-Nikitin et al., 2021; Huang et al., 2020). Conversely, elevated ROS levels in the tissue microenvironment can inhibit the interaction between BMAL1 and CLOCK proteins, disrupting transcription and the TTFL, which further exacerbates circadian disruption (Ranieri et al., 2015). (−)-Epigallocatechin-3-gallate (EGCG), a key component of green tea, exhibits strong antioxidant properties and has the potential to modulate the circadian clock to promote IVD health. EGCG has been shown to alleviate phase shifts induced by hydrogen peroxide (H_2_O_2_) and to regulate daily fluctuations in the transcription and protein expression levels of CR genes in a BMAL1-dependent manner. This suggests that EGCG may offer therapeutic benefits for mitigating oxidative stress and enhancing the health of IVDs (Mei et al., 2021; Krupkova et al., 2016).

Nuclear respiratory factor 2 (Nrf2) is a vital transcription factor that plays a significant role in regulating cellular responses to oxidative stress and maintaining redox homeostasis (Pekovic-Vaughan et al., 2014). Recent research has revealed a complex interplay between Nrf2 signaling and CRs, suggesting that Nrf2 not only responds to oxidative stress but also actively influences the regulation of circadian clock genes. Activation of Nrf2 can modify the expression and rhythmicity of circadian genes. For instance, chemical activation of Nrf2 has been shown to enhance the expression of core clock genes, resulting in increased circadian amplitude across various cell types (Wible et al., 2018). Additionally, Nrf2 is directly regulated by the circadian transcription factor BMAL1, particularly in lung and pancreatic tissues (Pekovic-Vaughan et al., 2014). BMAL1 binds to E-Box elements within the *Nrf* gene, regulating the rhythmic expression of Nrf2 and its downstream antioxidant stress proteins. This process mimics the transcription of antioxidant response elements (ARE) and other critical antioxidant proteins in accordance with CRs (Early et al., 2018; Zhang et al., 2023). In NPCs, the loss of BMAL1 leads to a decrease in the expression of Nrf2, which in turn results in increased levels of intracellular pro-inflammatory factors, heightened oxidative stress, increased apoptosis, and degradation of the ECM (Xiang et al., 2022). This interaction indicates that Nrf2 may act as a bridge between oxidative stress responses and circadian regulation, suggesting that the timing of Nrf2 activation is crucial for optimal cellular function and survival during stress conditions (Wible et al., 2018).

**FIGURE 3:**
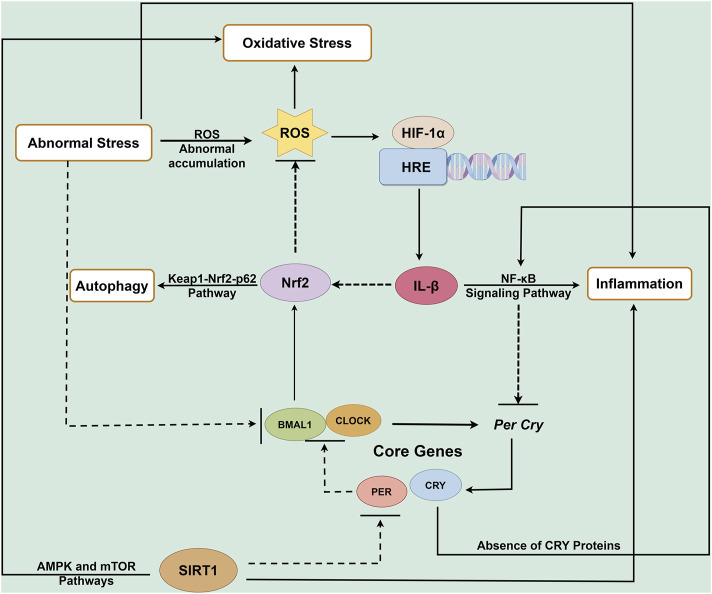
Circadian rhythm regulatory network in IDD. Many key molecules involved in the process of IDD dependent on the circadian clock. Circadian clock gene disruption leads to NF-κB overactivation (pro-inflammatory response), enhanced extracellular matrix degradation, Nrf2 suppression (oxidative damage), and impaired autophagy, collectively contributing to intervertebral disc degeneration. Disruption of circadian clock genes triggers NF-κB activation (inflammation), suppresses Nrf2 signaling (antioxidant response), promotes extracellular matrix degradation, and impairs autophagy, ultimately contributing to the development and progression of IDD. HRE: hypoxia response elements.

In summary, disruption of CR can link multiple pathways that promote IDD (Figure 3). Sirtuin 1 (SIRT1) is identified as a circadian deacetylase that plays a crucial role in regulating core clock components (Zhou et al., 2014). This NAD(+)-dependent enzyme functions as a histone deacetylase, with its activity influenced by the redox state of the NAD cofactor, which counteracts the activity of CLOCK. SIRT1 interacts with CLOCK-BMAL1 and PER2 in a circadian manner, facilitating the deacetylation and degradation of PER2. It is believed that SIRT1 and PER2 engage in a reciprocal negative regulation loop that modulates the circadian clock and influences aging, a relationship observed in both mouse and human hepatocytes (Wang et al., 2016). SIRT1 also plays a role in activating autophagy by regulating the AMPK and mTOR pathways (Ghosh et al., 2010; Packer, 2020). Research indicates that the deacetylation activity of SIRT1 can enhance the expression of autophagy-related genes, thereby increasing cellular autophagy levels (Ying et al., 2019). Conversely, inhibition of SIRT1 may lead to autophagy dysfunction, which can exacerbate inflammatory responses through the activation of Nuclear factor-κB (NF-κB) signaling (Takeda-Watanabe et al., 2012). Thus, SIRT1 may act as a bridge connecting the circadian clock, autophagy, redox state, and inflammation. Various pathways can further compound CR disruption through their interactions. For instance, excessive stress can lead to the abnormal accumulation of ROS and aberrant activation of the NF-κB signaling pathway, contributing to a senescent phenotype in NCPs (Li et al., 2017a). Additionally, abnormal mechanical tension can induce endoplasmic reticulum stress-mediated autophagy responses, ultimately leading to IDD (Chen et al., 2019).

### 4.2 The interaction between CRs and inflammation

Inflammation plays a crucial role in accelerating IDD (Li et al., 2017b). Various innate immune responses and functions are influenced by the time of day, and many inflammatory diseases are linked to dysfunctions in the molecular clocks of immune cells (Early et al., 2018). Disruption of biological rhythms can exacerbate inflammatory conditions. For instance, chronic sleep deprivation can disturb the biological clock, resulting in elevated levels of inflammatory factors, which is associated with an increased susceptibility to metabolic diseases such as diabetes and obesity, as well as cancer (Narasimamurthy et al., 2012). When examining the interaction between biological rhythms and inflammatory pathways, particularly the IL-1β-mediated NF-κB signaling pathway, a complex interplay has been noted (Dudek et al., 2017). This suggests that intervening in clock genes and their downstream target genes could be an effective strategy to mitigate inflammation-induced IDD.

#### 4.2.1 CRs regulates inflammation

The proper functioning of the biological clock is essential for regulating an appropriate level of inflammatory response (Spengler et al., 2012). NF-κB, a key transcription factor, governs various inflammatory processes, and its activity is influenced by the biological clock. Research has demonstrated that BMAL1 and CLOCK can modulate the transcriptional activity of NF-κB, thereby playing a significant role in immune responses (Spengler et al., 2012). For instance, IL-1β can activate the NF-κB signaling pathway, promoting the expression of inflammatory factors. However, this process may vary depending on CRs. In Clock-deficient cells, there is a notable reduction in NF-κB activation levels in response to immune stimulation (Spengler et al., 2012). Additionally, the deletion of the BMAL1 gene leads to dysregulated glycolysis in mouse macrophages, increased oxidative stress, and heightened expression of the cytokine IL-1β, which further exacerbates inflammation (Early et al., 2018). Moreover, the absence of CRY proteins may remove their inhibitory effect on cAMP production, resulting in elevated cAMP levels and increased protein kinase A (PKA) activation. This cascade can lead to NF-κB activation through the phosphorylation of p65 at serine 276, ultimately triggering a chronic inflammatory state (Narasimamurthy et al., 2012).

#### 4.2.2 Inflammation regulates CRs

The activation of NF-κB significantly influences the transcriptional activity of core components of the circadian clock. In the presence of inflammatory stimuli, NF-κB activation leads to a marked suppression of clock repressors, including the *Per, Cry,* and *Rev-erb* genes. Additionally, NF-κB activation causes the relocation of CLOCK/BMAL1 components across the genome to sites that are bound by NF-κB (Hong et al., 2018). In the context of osteoarthritis, studies have shown that the expression of BMAL1 in human chondrocytes decreases as inflammation severity increases (Dudek et al., 2016). Guo B and colleagues utilized two luciferase-based reporter mouse models to monitor circadian gene activity in real time. The first, PER2LUC, is a transgenic model in which the *Per2* gene promoter drives the expression of a firefly luciferase enzyme fused to the PER2 protein. This system enables continuous bioluminescence imaging, as oscillations in PER2 levels reliably reflect the function of the core circadian clock. The second model, Cry1-luc, uses the *Cry1* gene promoter to regulate luciferase expression. This reporter allows real-time tracking of *Cry1* transcriptional activity in cartilage tissue, offering a sensitive readout of clock gene activation driven by E-box elements. Their study demonstrated that activation of the NF-κB signaling pathway—particularly through interleukin-1β (IL-1β)—disrupts normal CRs by suppressing the expression of both Cry1-luc and PER2LUC (Guo et al., 2015). This disruption of the circadian clock is a critical factor associated with inflammation-related autophagy in IDD. IL-1β not only impairs the normal molecular functions of clock-related components but also plays a role in various pathological processes during IDD, such as inflammatory responses, matrix degradation, angiogenesis, nerve innervation, apoptosis, oxidative stress, and cellular aging. Furthermore, IL-1β activates protective autophagy in IVDs (Yang et al., 2015). It has been noted that IL-1β can inhibit the expression of *Nrf2*, which is implicated in the induction of IDD. However, using the Nrf2 agonist sulforaphane can promote oxidative stress-induced autophagy in NCPs through the Keap1/Nrf2/p62 feedback loop, thereby alleviating the degeneration phenotype of the disc (Tang Z. et al., 2019).

### 4.3 The correlation between chronotypes and the incidence of IDD

Chronotype refers to the temporal characteristics associated with an individual’s activity rhythm and sleep-wake cycle, reflecting their actual activity tendencies and responses to external schedules. Chronotypes are typically classified into three categories: morning type, intermediate type, and evening type (Vitale and Weydahl, 2017). Morning types prefer to engage in activities earlier in the day, while evening types tend to be more active later, with the majority of individuals falling into the intermediate category. Morning chronotypes are often better aligned with societal norms and social interactions, whereas evening types are more prone to experiencing CR disorders. Factors such as age, gender, and genetic variations in certain genes influence chronotypes, with some genes linked to longer or shorter intrinsic CRs. Chronotypes also affect how well individuals align with external environments and the phases of their internal biological clocks. Research has shown that different chronotypes are associated with various health issues, including cardiovascular diseases, dry eye syndrome, depressive moods, and rheumatoid arthritis (Wang et al., 2022; O’Leary et al., 2018). However, the relationship between chronotypes and ID remains unclear. Current hypotheses suggest that individuals with a stronger tendency towards evening types, especially those engaged in shift work or night work, may have a higher risk of IDD (Ghotbi et al., 2023; McMahon et al., 2019). The underlying mechanism is thought to be closely linked to social jetlag, which describes the misalignment between an individual’s endogenous circadian rhythm and externally imposed social schedules (Haraguchi et al., 2021). Individuals with an evening chronotype (“owls”) exhibit heightened social jetlag due to forced alignment with early societal demands (e.g., work/school start times), resulting in prolonged circadian disruption, consequently, more pronounced rhythm disruptions (Takahashi et al., 2018). This misalignment correlates with systemic consequences such as metabolic dysregulation (Arab et al., 2024), low-grade inflammation (Haspel et al., 2020), and impaired tissue repair—all pathways implicated in IVD degeneration.

## 5 The application of circadian rhythms in the management of intervertebral disc diseases

Patients with degenerative lumbar disc disease often experience mechanical low back pain, particularly after engaging in weight-bearing activities. The extrusion of disc material, especially the nucleus pulposus, can trigger inflammatory responses and irritate nearby nerve roots. As the degeneration progresses, physical narrowing may lead to symptoms such as neurogenic claudication, radiculopathy, and cauda equina syndrome (Wu et al., 2020). For patients who do not exhibit neurological dysfunction, the primary focus of treatment is pain management. Most patients benefit from conservative treatment options, which are typically the first line of approach. However, for those with persistent symptoms that do not improve with conservative measures, surgical interventions may be recommended. These can include minimally invasive procedures or open spinal fusion (Xin et al., 2022). Recent research has shed light on the effects of CR disruption and the circadian regulatory system, opening new avenues for the treatment of IDD.

### 5.1 Non-surgical treatment

Non-surgical treatment for degenerative lumbar disc disease is generally divided into two categories: drug therapy and non-drug therapy. Non-steroidal anti-inflammatory drugs (NSAIDs) are the primary medications used to alleviate pain symptoms by reducing inflammation in the pain pathways. Among these, specific COX-2 inhibitors are known to have fewer gastrointestinal side effects compared to non-selective COX inhibitors (Hahne et al., 2010). Common NSAIDs include aspirin and indomethacin, among others. Aspirin is most effective when taken in the morning, approximately half an hour after breakfast, as this timing allows for higher blood concentrations, resulting in a longer duration of action and improved bioavailability. In contrast, taking aspirin in the evening leads to lower blood levels. Therefore, it is recommended to take an adequate dose in the morning. Similarly, indomethacin is best administered at 7 a.m., which facilitates the rapid achievement of effective plasma concentrations while minimizing adverse reactions. The judicious use of non-steroidal analgesics not only enhances postoperative pain relief but also significantly reduces the time required for gas passage and bowel movements following abdominal surgery (Milne et al., 2018).

### 5.2 Surgical intervention

CRs significantly influence the sensitivity to and duration of action of general anesthetics. General anesthesia itself can be an independent risk factor for postoperative sleep disturbances, which in turn affects recovery and sleep quality in patients. A clinical trial involving patients undergoing elective laparoscopic abdominal surgery under general anesthesia revealed that those who had surgery in the evening (between 6:00 p.m. and 11:00 p.m.) were more likely to experience postoperative sleep disturbances, increased pain, and other adverse events compared to those who had surgery during the day (between 8:00 a.m. and 12:00 p.m.). This phenomenon may be attributed to the fact that propofol, a common anesthetic, primarily induces anesthesia through GABA A receptors, which are more active at night and play a crucial role in regulating sleep (Song et al., 2020). Moreover, both surgery and anesthesia can disrupt the body’s circadian timing system. The timing of elective surgeries can impact clinical outcomes, as the physiological cycles of patients and the performance of the surgical team are affected by CRs. Research indicates that elective surgeries performed after regular work hours are associated with higher risks of mortality and morbidity compared to those conducted during the day (Meewisse et al., 2024). This increased risk may be linked to surgeon fatigue and reduced resource availability at night. Additionally, some studies have noted a higher incidence of thromboembolic events and decreased blood loss during morning surgeries. This observation aligns with earlier findings that suggest diurnal rhythms influence platelet activation (Tofler et al., 1987) and the production of clotting factors (Montagnana et al., 2009). Consequently, it is recommended that surgeries expected to involve significant blood loss be scheduled for the morning, while patients at high risk for thromboembolic complications should ideally have their procedures in the afternoon (Meewisse et al., 2024).

### 5.3 The relationship between CRs and postoperative recovery and outcomes

Post-operative complications are a significant contributor to all-cause mortality, ranking as the third greatest cause (Nepogodiev et al., 2019). Common issues following spinal surgery include postoperative intestinal paralysis and wound pain. Such surgeries can disrupt the CR associated with bowel movements. However, the appropriate use of Mel, serotonin (5-HT) receptor agonists, and NSAIDs during the perioperative period can help restore this rhythm (Duboc et al., 2020). Research by Baradari et al. indicated that patients who took 5 mg of Mel 1 hour before surgery reported significantly reduced pain intensity following lumbar laminectomy and discectomy (Baradari et al., 2022). Furthermore, enhancing patients’ preoperative functional capacity through CR prehabilitation can mitigate the risk and severity of postoperative complications (Stretton et al., 2024). Disruption of the circadian clock due to irregular feeding and sleeping patterns is closely linked to various adverse health outcomes (Allada and Bass, 2021). Time-restricted eating (TRE), which involves a limited eating window of 8–12 h (or shorter) with consistent meal times but without caloric restriction, has shown promise in improving health (Wilkinson et al., 2020). Establishing a consistent sleep window based on an individual’s internal physiological clock can help slow the progression of obesity, diabetes, and cardiometabolic diseases (Allada and Bass, 2021; Chaput et al., 2020). Given this context, patients with metabolic conditions such as hypertension, obesity, and diabetes may benefit from preoperative education on CRs, including strategies like time-restricted eating and maintaining a regular day-night cycle.

## 6 Emerging therapies

### 6.1 Pulsed electromagnetic field (PEMF)

Non-drug therapies, particularly physical therapy and rehabilitation programs, play a crucial role in helping patients regain normal functional activities and preventing further injuries. One such non-invasive treatment gaining attention is PEMF therapy. This approach has become increasingly popular in recent years due to its low cost and effectiveness in treating musculoskeletal diseases, including osteoporosis (Wang et al., 2019), osteoarthritis (Yang et al., 2020) and spinal fusion (Akhter et al., 2020). PEMF therapy enhances bone health by influencing various cellular processes. It promotes the proliferation and differentiation of osteoblasts, inhibits the formation of osteoclasts, and affects the activity of bone marrow mesenchymal stem cells (BMSCs) and osteocytes (Wang et al., 2019; Esmail et al., 2012). Recent studies have also indicated that PEMF can alleviate discogenic back pain (Zheng et al., 2022a; Tang X. et al., 2019). For instance, research by Miller et al. demonstrated that applying electromagnetic field interventions to human NPCs led to a reduction in the expression of inflammatory factors and catabolic products induced by IL-1α, thereby promoting anabolic metabolism (Miller et al., 2016). Additionally, Zheng et al. found that electromagnetic field interventions could upregulate the expression of EC-related genes in degenerated NPCs. This intervention also increased the expression of SIRT1, a protein associated with cellular health, and promoted autophagy in these degenerated cells (Zheng et al., 2022b).

The timing of treatment intervals can significantly influence therapeutic outcomes, as demonstrated in various studies. For instance, a study exploring the combination of PEM therapy and dexamethasone found that PEMF could counteract the inhibitory effects of dexamethasone on osteoblast proliferation, particularly during the early treatment phase (Esmail et al., 2012). Additionally, research on photobiomodulation (PBM) treatment intervals revealed that longer intervals resulted in significantly higher cytochrome c oxidase (CCO) activity in specific brain regions compared to shorter intervals (Arias et al., 2020). Despite these insights, the optimal timing for PEMF application remains underexplored. In the context of peripheral nerve injury, daytime PEMF stimulation has been shown to yield more effective outcomes for nerve regeneration and functional recovery than nighttime stimulation (Zhu et al., 2017). Similarly, in osteoporosis treatment, early-phase PEMF stimulation is more beneficial than stimulation applied later (Zhou et al., 2017). This evidence suggests that the CR may play a crucial role in the therapeutic effectiveness of PEMF in treating IDD. Notably, studies in rats indicated that dynamic PEMF (DPEMF) stimulation is more effective than static PEMF (NPEMF) stimulation in alleviating IDD (Zheng et al., 2022a). Given the differences in sleep-wake cycles and circadian activity between rats and humans, with a roughly 12-h offset, it is speculated that nighttime PEMF therapy may be more advantageous for humans in alleviating IDD compared to daytime therapy.

### 6.2 Pharmacotherapy targeting CRs for the treatment of IDD

Chronobiotic drugs like Mel are utilized to help restore disrupted CRs. Mel is an endocrine hormone produced primarily in the pineal gland and mitochondria, and it has shown promise in treating various CR-related sleep disorders, including jet lag, shift work issues, and insomnia (Williams et al., 2016). Mel also holds potential as a chronotherapy agent for age-related IDD. It operates through several mechanisms, such as regulating oxidative stress and inflammation, reducing the effects of aging, promoting autophagy, and modulating CRs (Kaye et al., 2024). In NPCs, Mel has been found to reduce the aggregation of inflammatory cells and the release of inflammatory factors, which enhances the remodeling of the ECM within the nucleus pulposus (Zhang et al., 2019). Furthermore, in the A, Mel can lower ROS levels and inhibit the activity of the NF-κB signaling pathway, thereby promoting autophagy and slowing cellular aging (Li et al., 2021). Additionally, Mel enhances the survival and function of nucleus pulposus cells by activating the ERK1/2 signaling pathway. This process not only boosts cell vitality but also inhibits cell cycle arrest and apoptosis, thereby slowing down disc degeneration (Ge et al., 2019). By stimulating this pathway, Mel can improve the survival and functionality of NPCs, suggesting its potential as a therapeutic option for IDD and related conditions (Kaye et al., 2024).

The therapeutic strategies aimed at modulating cellular CRs with drugs face challenges due to the short half-lives of these medications and the complexities of the *in vivo* microenvironment (Hwang et al., 2021). Despite these limitations, Mel injection therapy has demonstrated significant potential in rat models, similar to the effects seen with corticosteroid injections and platelet-rich plasma (PRP) treatments for knee joints (Wu et al., 2023). However, while Mel has shown promising therapeutic effects in animal studies, direct clinical trials involving human patients have not yet been conducted. In the field of tissue-engineered materials, research has focused on exploring the effectiveness of CR-modulating drugs for clinical applications. This includes optimizing the timing and dosage of drug interventions, as well as refining drug delivery systems to enhance their effectiveness. One innovative approach involves the use of polyvinyl alcohol (PVA) microspheres that are crosslinked with modified phenylboronic acid (PBA) and contain Mel liposomes. This formulation has the capability to regulate the expression of core circadian clock genes by activating the PI3K-AKT signaling pathway in NPCs. This activation helps to remodel the intrinsic circadian clock and promotes the synthesis of the ECM (Chen et al., 2023).

Another category of substances that influence CRs includes natural compounds. For instance, consuming caffeine in the evening has been found to delay the human circadian Mel rhythm. Additionally, chronic caffeine intake can extend the circadian period of molecular oscillations *in vitro* (Burke et al., 2015). Resveratrol, a natural polyphenol, has also been shown to affect CRs by inducing the SIRT1 protein and altering the rhythmic expression of key circadian genes such as *Clock*, *Bmal1*, and *Per2* (Spaleniak and Cuendet, 2023). Furthermore, the intake of resveratrol has been associated with improvements in the rhythmicity of blood glucose, insulin, and leptin levels in mice that are subjected to a high-fat diet, thereby enhancing their overall metabolic status (Sun et al., 2015) (Table 1).

**TABLE 1:** Pharmacotherapy targeting CRs for the treatment of IDD.

Chronobiotic drugs name	Drug properties	Pharmacological action
Melatonin (Mel)	Endocrine hormone	Adjust circadian rhythm
Caffeine	Central Nervous System Stimulant	Delayed the circadian rhythm of melatonin
(-)-Epigallocatechin-3-gallate (EGCG)	Polyphenol	Protects IVD Cells from Oxidative Stress
Resveratrol	Polyphenol	Alter the expression of clock genes and improve endocrine and metabolic states

Molecularly targeted drugs name	Target	Pharmacological action
Nobiletin	Activation of BMAL1 and RORα	Clock amplitude enhancement and enhanced metabolism
KL001 and KS15	Stabilize CRY and inhibit BMAL1	Affect exercise rhythm
GSK4112	Activation of REV-ERBα	Inhibit the release of inflammatory factors (IL-6, CXCL11, and CCL2)
SR9009 and SR9011	Activation of REV⁃ERBa	Improve metabolic homeostasis in obese individuals and enhance mitochondrial content in skeletal muscle
Cystathionine β-synthase (CBS)	Binding to CRY1	Augments CRY1-mediated repression of the CLOCK/BMAL1 complex and shortens circadian period

### 6.3 Circadian clock molecular targeted drugs

Candidate targeted drug molecules for clock molecules. Nobiletin, a natural polymethoxylated flavone, can directly activate RORa, enhancing the oscillation of the rhythmic protein BMAL1 and boosting energy metabolism in obese individuals (He et al., 2016). Nobiletin and SR8278 can effectively activate the expression of BMAL1 and inhibit the cell proliferation induced by IL-β in osteoarthritis models (He et al., 2022). In male *Drosophila melanogaster*, KL001 and KS15 inhibit the activity of BMAL1 by stabilizing CRY1, thereby extending lifespan and affecting locomotor rhythms (Solovev et al., 2021). KL001 stabilizes CRYs proteins by competing with the carboxy-terminal end of FBXL3 for binding to the FAD-binding pocket, thereby reducing CRYs degradation and regulating the circadian period (Nangle et al., 2013). The REV-ERBa agonist GSK4112 can inhibit the release of inflammatory factors (IL-6, CXCL11, and CCL2) induced by endotoxins in the THP1 cell line (Gibbs et al., 2012). The REV-ERBa agonists SR9009 and SR9011 improve metabolic homeostasis in obese individuals, enhance mitochondrial content in skeletal muscle, and increase exercise endurance (Solt et al., 2012; Woldt et al., 2013). In cells, cystathionine β-synthase (CBS), a central enzyme in one-carbon metabolism, augments CRY1-mediated repression of the CLOCK/BMAL1 complex and shortens circadian period (Cal-Kayitmazbatir et al., 2021) (Table.1).

### 6.4 Potential of personalized medicine based on CR characteristics

CRs play a crucial role in influencing treatment responses and outcomes across a range of medical conditions. By understanding how these biological rhythms interact with drug interventions, researchers have been able to develop chronotherapy. This therapeutic approach aims to optimize treatment effects by timing drug administration to align with the body’s natural CRs (Ribeiro et al., 2021). Chronotherapy has been clinically implemented in various disease areas, including hypertension, high cholesterol, bronchial asthma, and certain types of cancer (Allada and Bass, 2021; Man et al., 2021). This approach is particularly significant for conditions like rheumatoid arthritis (RA), where symptoms such as joint pain and stiffness tend to worsen in the morning due to the CRs of cytokine and hormone levels. Research has demonstrated that timing glucocorticoid therapy to coincide with the nocturnal increase in inflammatory markers can lead to substantial improvements in patient outcomes, significantly reducing morning stiffness and pain compared to administering the same dose at other times of the day (Levi et al., 1985; Buttgereit et al., 2008; Buttgereit et al., 2015). In patients with bipolar disorder, the circadian rhythmic characteristics of time perception have been linked to treatment responses, indicating that aligning therapeutic interventions with the patient’s CRs may enhance treatment outcomes (Yoshiike et al., 2020). In the realm of cancer treatment, CRs are also crucial. Chronotherapeutics, which involve administering chemotherapy at specific times to align with the body’s biological clock, have shown improved tolerability and efficacy. Studies suggest that administering anti-cancer medications in accordance with CRs can enhance treatment effectiveness and reduce side effects, thereby improving overall patient care (Scully et al., 2011).

The integration of chronobiology into therapeutic strategies offers a promising avenue for enhancing treatment outcomes across various medical conditions. By taking into account the relationship between interventions and patients’ CRs, healthcare providers have the potential to improve both the efficacy and safety of treatments, ultimately leading to better overall health outcomes. However, the concept of “timing” in treatments, including medication and surgery, is often overlooked in clinical practices related to IDD. Currently, chronotherapy has not been widely applied in the treatment of IDD. Genome-wide studies have shown that circadian sampling in rodents, non-human primates, and human tissues indicates that over 80% of FDA-approved drug targets exhibit daily rhythms (Zhang et al., 2014; Ruben et al., 2018). Despite this, there are very few drugs available that can restore circadian phase specifically for the treatment of IDD. Chronotype is a central determinant in the personalization of chronotherapy. Variations in chronotype—typically classified as “larks” (morning types) and “owls” (evening types)—can lead to circadian phase shifts of up to 4 hours, which may significantly alter the optimal therapeutic window.

In the context of IDD treatment, the goal of chronotherapy would be to synchronize the timing of surgical interventions, drug administration, and postoperative rehabilitation exercises with CRs. This synchronization could potentially minimize hospital stays and enhance the quality of treatment. However, employing CRs to inform clinical treatment presents several challenges. These include effectively monitoring patients’ circadian cycles, addressing biases that may arise from rhythmic heterogeneity among patients, and managing the complexity of the IVD, which is a multilayered composite tissue.

## 7 Conclusion and future perspectives

Both clinical data analysis and animal model validation, along with investigations into molecular mechanisms at the cellular level, suggest that CRs play a significant role in the regulation of IDD. The evidence indicates that the normal functioning of CRs is beneficial for understanding the pathophysiology of IDD and may also contribute positively to treatment strategies. In the context of IDD, recent studies have highlighted the role of a novel microRNA, hsa-let-7f-1-3p, in regulating CRs and the function of IVD cells. However, there remains a significant gap in research regarding key biomarkers and therapeutic targets linked to CRs in IDD (Mei et al., 2022). Gene expression analysis of IVD cells has enabled researchers to identify critical genes associated with autophagy, such as *CTSD*, *VEGFA*, and *BAX*, as well as genes related to oxidative stress and immune infiltration, including PPIA and PXN. The expression levels of these genes may correlate with the extent of degeneration and the sensitivity to pain in the IVD (Wang et al., 2023; Xiang et al., 2024). Additionally, LncRNA HCG18 has been found to promote inflammation and apoptosis during the degeneration process through the miR-495-3p/FSTL1 axis (Luo et al., 2024). Given the established connections between CRs, autophagy, inflammation, and oxidative stress, targeting these genes through specific interventions may enhance circadian regulation in the IVD, potentially leading to improved treatment outcomes for IDD.

How CRs control cellular behavior, metabolism, and signal transduction during the process of IDD still requires further exploration. Currently, research on the impact of biological clocks on IVDs mainly focuses on the phenotypic changes caused by the knockout or mutation of clock molecules (*Bmal1, Per2, and Dbp*). It remains unclear what factors can induce changes in clock molecules and the specific downstream mechanisms through which these changes can affect IVD homeostasis *via* signaling pathways.

Future research directions should include: 1. Impact of External Factors: Investigating whether external environmental factors such as light exposure, dietary patterns, physical activity, and regular sleep can help sustain or restore the CR in IVDs; 2. Interactions Among Clock Molecules: Exploring how different clock molecules interact with one another in their physiological regulatory roles within IVDs and their potential associations with the onset of degenerative diseases; 3. Rhythmic Communication and Synergistic Effects: Although some regulatory pathways of the molecular clock system in IVD cells have been identified, the mechanisms of rhythmic communication between cells and the synergistic effects of these networks remain poorly understood; 3. Assessment Indicators for CR Disorders: There is a notable lack of standardized and effective assessment indicators for diagnosing CR disorders in clinical settings. Developing reliable metrics to evaluate these disorders is essential for advancing “chronotherapy” into clinical practice, allowing for tailored treatment approaches based on individual circadian profiles. Addressing these issues will be crucial for enhancing our understanding of CRs in IVD health and degeneration, ultimately paving the way for innovative therapeutic strategies.
